# Clinically meaningful eGFR slope as a surrogate endpoint differs across CKD stages and slope evaluation periods: the CKD-JAC study

**DOI:** 10.1093/ckj/sfae398

**Published:** 2025-01-13

**Authors:** Takahiro Imaizumi, Hirotaka Komaba, Takayuki Hamano, Masaomi Nangaku, Kenta Murotani, Takeshi Hasegawa, Naohiko Fujii, Kosaku Nitta, Yoshitaka Isaka, Takashi Wada, Shoichi Maruyama, Masafumi Fukagawa

**Affiliations:** Department of Advanced Medicine, Nagoya University Hospital, Nagoya, Japan; Department of Nephrology, Nagoya University Graduate School of Medicine, Nagoya, Japan; Division of Nephrology, Endocrinology and Metabolism, Tokai University School of Medicine, Isehara, Japan; Department of Nephrology, Nagoya City University Graduate School of Medicine, Nagoya, Japan; Department of Nephrology, Graduate School of Medicine, the University of Osaka, Suita, Japan; Division of Nephrology and Endocrinology, the University of Tokyo Hospital, Tokyo, Japan; School of Medical Technology, Kurume University, Kurume, Japan; Biostatistics Center, Kurume University, Kurume, Japan; Institute of Clinical Epidemiology; Department of Hygiene, Public Health, and Preventive Medicine, Graduate School of Medicine; Department of Nephrology, Graduate School of Medicine; Showa University Research Administration Center, Showa University, Tokyo, Japan; Center for Innovative Research for Communities and Clinical Excellence, Fukushima Medical University, Fukushima, Japan; Medical and Research Center for Nephrology and Transplantation, Hyogo Prefectural Nishinomiya Hospital, Nishinomiya, Japan; Department of Nephrology, Tokyo Women's Medical University, Tokyo, Japan; Department of Nephrology, Graduate School of Medicine, the University of Osaka, Suita, Japan; Department of Nephrology and Rheumatology, Kanazawa University, Kanazawa, Japan; Department of Nephrology, Nagoya University Graduate School of Medicine, Nagoya, Japan; Division of Nephrology, Endocrinology and Metabolism, Tokai University School of Medicine, Isehara, Japan

**Keywords:** chronic kidney disease, clinical epidemiology, eGFR slope, kidney failure, surrogate endpoint

## Abstract

**Background:**

The slope of estimated glomerular filtration rate (eGFR) is a promising surrogate endpoint in patients with chronic kidney disease (CKD). However, current evidence is mainly derived from Western populations with CKD stages 1–3. In addition, stage-by-stage analysis has never been formally performed.

**Methods:**

We analyzed data from the Chronic Kidney Disease Japan Cohort Study, which included a large proportion of patients with CKD stages 4 and 5. We estimated eGFR slopes over three evaluation periods (0.5, 1, and 2 years) using mixed effects models and examined their associations with kidney failure with replacement therapy across CKD stages.

**Results:**

Of 2713 patients with an available 1-year eGFR slope, 985 subsequently initiated kidney replacement therapy. Overall, a slower eGFR decline was strongly associated with a lower risk of subsequent kidney failure with replacement therapy. The association was pronounced with higher baseline CKD stages and attenuated with shorter evaluation periods. The estimated deceleration in eGFR decline over 1 year associated with a 20% lower risk of subsequent kidney failure with replacement therapy was 1.91 (1.60–2.37), 1.12 (1.00–1.28), and 1.06 (0.81–1.60) ml/min/1.73 m^2^ per year in patients with CKD stages 3, 4, and 5, respectively.

**Conclusion:**

Our results support the potential of eGFR slope as a surrogate across different stages of CKD in Asians and suggest that a shorter evaluation period than 2 years may be feasible for patients with late-stage CKD. Our findings provide valuable insights for the future design of clinical trials in CKD patients, especially those with more advanced CKD.

## Graphical Abstract

**Graphical Abstract:**
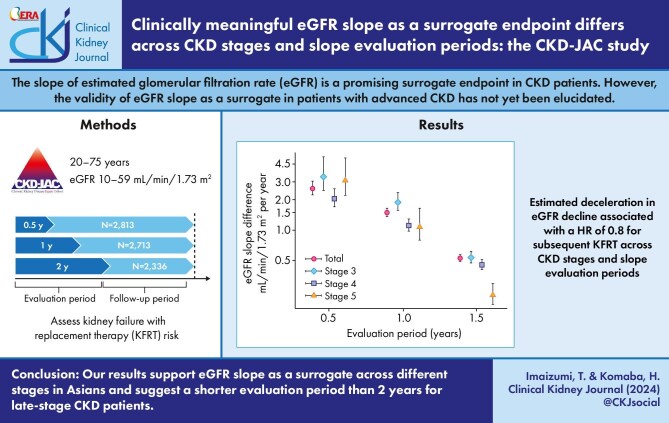

KEY LEARNING POINTS **What was known**:

- The slope of estimated glomerular filtration rate (eGFR) during 2 years is a promising surrogate endpoint in patients with chronic kidney disease (CKD)
- The validity of eGFR slope has been primarily investigated in Western populations with mild-to-moderate CKD.
- However, stage-by-stage analysis has never been formally performed.

**This study adds**:

- We demonstrated associations between eGFR slope and the risk of kidney failure across CKD stages in Japanese CKD patients including advanced CKD.
- The clinically meaningful eGFR slope over 1 year associated with a 20% lower risk of subsequent kidney failure with replacement therapy was smaller in more advanced stages.

**Potential impact**:

- Our results support the potential of eGFR slope as a surrogate across different stages of CKD in Asians and suggest a shorter evaluation period than 2 years for late-stage CKD patients.
- Our findings provide valuable insights for future design of clinical trials in CKD patients, especially those with more advanced CKD.

## INTRODUCTION

Establishing reliable surrogate endpoints is an important issue for the efficient design of clinical trials in patients with chronic kidney disease (CKD). Although a 30–40% decline in estimated glomerular filtration rate (eGFR) was established as a surrogate endpoint [1–4], recent advances in CKD management have led to only a limited number of patients reaching these endpoints [4, 5]. To address this, a scientific workshop in the United States and Europe proposed that a slower decline in eGFR by 0.5–1.0 ml/min/1.73 m^2^ per year can ensure a reliable treatment effect on long-term clinical outcomes [6].

However, the validity of eGFR slope as a surrogate endpoint has primarily been studied in individuals with mild-to-moderate CKD. According to the landmark CKD Prognosis Consortium (CKD-PC) meta-analysis [5], the mean eGFR was 47 ml/min/1.73 m^2^ even in the lower eGFR subgroup (<60 ml/min/1.73 m^2^), suggesting that only a small proportion of patients had CKD stage 4 or 5. Although recent clinical trials have enrolled participants with more advanced CKD and examined the effect of medication on eGFR slope [7–11], the validity of the eGFR slope in advanced CKD has not yet been established.

The rationale for using eGFR slope may be perceived as limited in patients with advanced CKD, as they are expected to initiate kidney replacement therapy (KRT) soon. This limitation is particularly relevant for a subset of patients with rapidly progressive CKD and those in regions where KRT is initiated relatively early with preserved kidney function [12]. However, the use of the eGFR slope in advanced CKD could become feasible even in such populations if novel drugs capable of halting CKD progression become available. Therefore, it is crucial to investigate the validity of eGFR slope also in patients with advanced CKD.

Another important consideration when using eGFR slope as a surrogate is determining the optimal evaluation period needed for accurately estimating the eGFR slope. Whereas shorter evaluation periods allow for clinical trials with shorter follow-up durations, they may require a larger sample size due to potentially less-precise estimates of eGFR slope. Previous studies have assessed the validity of the eGFR slope calculated over periods ranging from 1 to 3 years [5, 9]. However, the validity of estimating the eGFR slope within shorter evaluation periods has not been thoroughly explored.

To address these knowledge gaps, we conducted a comprehensive analysis using data from the Chronic Kidney Disease Japan Cohort (CKD-JAC) Study, which included a large number of patients with CKD stages 4 and 5. Our study had two primary objectives. First, we aimed to assess the validity of the eGFR slope across different stages of CKD and different slope evaluation periods (0.5, 1, and 2 years). Second, we sought to estimate the clinically meaningful difference in eGFR slope at each CKD stage.

## MATERIALS AND METHODS

### Source of data and participants

The source of data was the CKD-JAC Study, a multicenter, prospective observational cohort of 2966 participants across Japan. Patients aged 20–75 years with an eGFR of 10–59 ml/min/1.73 m^2^ were enrolled. Details of the study are described elsewhere [13].

**Figure 1:**
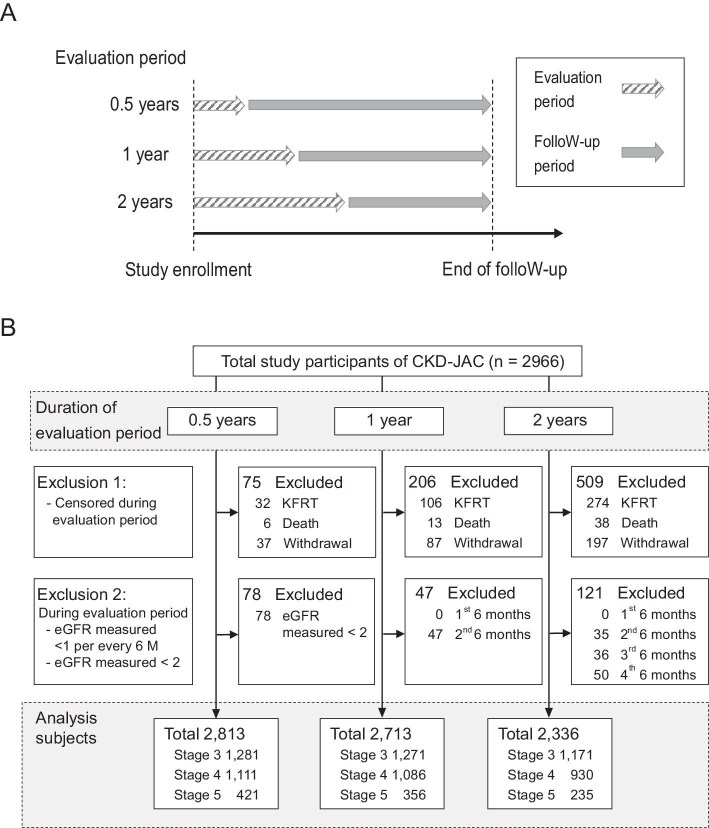
Study design and flow diagram. **A. Study design.** eGFR slopes were estimated during the evaluation periods of 0.5, 1, and 2 years, and covariate data were collected at study enrollment. The median [IQR] follow-up periods were 5.1 [2.5–9.3], 5.0 [2.4– 8.9], and 4.9 [2.1–8.0] years since the end of the 0.5, 1, and 2-year evaluation periods, respectively. The primary analysis was conducted using a 1-year evaluation cohort. **B. Flow diagram.** Participants whose eGFR was measured at least once every 6 months during the evaluation period were included. After excluding those who developed KFRT, died, or withdrew from the study within the evaluation period, 2813, 2713, and 2336 participants remained in the study. Abbreviations: eGFR, estimated glomerular filtration rate; KFRT, kidney failure with replacement therapy.

We first analyzed the whole cohort of patients in the CKD-JAC Study to examine the cumulative incidence of a composite of a 30% or 40% decline in eGFR and kidney failure with replacement therapy (KFRT) and the incidence of KFRT stratified by the baseline CKD stage (Supplementary Material). We then examined the association between the eGFR slopes and subsequent KFRT using three different evaluation periods (0.5, 1, or 2 years) during which eGFR slopes were estimated. The overview of the study design and the flow diagram are shown in Fig. 1. Study participants who reached a censoring event during the evaluation period and those who did not have eGFR measurements at least twice during the evaluation period and once every 6 months were excluded from the analysis. In this study, we considered 1-year slopes as the primary exposure. As recommended by the KDIGO CKD guideline [14], we primarily used an eGFR equation best tailored to the Japanese population [15]. The definitions of the covariates are described in Supplementary Methods.

### Follow-up and censoring events

The CKD-JAC Study began enrolling participants in April 2007 through December 2008, and followed them through June 30, 2018. Cardiovascular disease (CVD) events, all-cause death, and KFRT were recorded. Follow-up was censored at death, KFRT, transfer to other facilities, refusal to participate in the study, or administrative censoring (March 31, 2013 for one site and June 30, 2018 for the others). For participants who initiated KRT, we identified the last available eGFR values measured within up to 6 months before KRT initiation.

### Statistical analysis

The slope of eGFR was estimated using linear mixed models with an unstructured variance‒covariance matrix, random intercept, and random slope for each individual [16]. We also estimated eGFR slope by using the ordinary least squares method. We also used another surrogate endpoint, the percent decline in eGFR [3, 4, 17].

Descriptive data are expressed as the mean ± SD and median [interquartile range (IQR)] for normally and non-normally distributed continuous variables, respectively. Participants were divided into quartiles based on the 1-year eGFR slope: <−3.7 ml/min/1.73 m^2^ per year; −3.7 to −1.8 ml/min/1.73 m^2^ per year; −1.8 to 0 ml/min/1.73 m^2^ per year; and >0 ml/min/1.73 m^2^ per year.

Event-free survival in the whole cohort was calculated using the cumulative incidence function. Cox proportional hazards models were adjusted for age, sex, baseline eGFR, systolic blood pressure, history of CVD, smoking status, and total cholesterol [5] and stratified by facility. Fine–Gray models were employed for sensitivity analysis with the same variables adjusted. We performed several additional analyses, detailed in Supplementary Methods.

## RESULTS

### Baseline characteristics and kidney outcomes in the whole cohort

Patients with more advanced CKD were older, had higher urinary albumin–creatinine ratio (UACR) and blood pressure, and were more likely to have diabetes mellitus (DM; Table S1). Of the 2966 total CKD-JAC Study participants, 1097 initiated KRT. The incidence of KFRT and the composite of a 30% decline in eGFR and KFRT increased with higher baseline CKD stage (Fig. S1 and Table S2); however, even among the 465 patients with stage 5 CKD, only 85 (18%) initiated KRT within 1 year, and 173 (37%) initiated KRT within 2 years. Of the 1097 patients who developed KFRT, the last recorded eGFR value within 6 months before KRT initiation in 790 individuals was a median [IQR] of 5.2 [4.8–7.2] ml/min/1.73 m^2^.

### Baseline characteristics of study participants with 1-year evaluation period

Of the 2713 participants included in the 1-year eGFR slope analysis, 25% had a rapid eGFR decline (slope <−3.7 ml/min/1.73 m^2^ per year), 26% had a positive change (slope ≥0 ml/min/1.73 m^2^ per year), and the remaining 49% had a slower decline over the 1-year evaluation period (Table 1). Individuals with a rapid eGFR decline tended to have a worse risk profile (higher prevalence of albuminuria, history of CVD, current smoking status, and DM as a cause of kidney disease, and poorer blood pressure control) than did those with a slower decline or a positive slope. Patients with a rapid eGFR decline had more frequent eGFR measurements.

**Table 1:** Baseline characteristics by eGFR slope in the 1-year evaluation cohort.

	*N*	Total(2713)	<−3.7(678)	≥−3.7 to <−1.8(683)	≥−1.8 to <0(649)	≥0(703)
Age, years	2713	60 (11)	60 (12)	60 (11)	62 (11)	60 (12)
Male	2713	1671 (61.6)	431 (63.6)	411 (60.2)	382 (58.9)	447 (63.6)
eGFR, ml/min/1.73 m^2^	2713	29 (12)	30 (12)	26 (12)	27 (12)	34 (11)
CKD stage	2713					
3		1271 (46.8)	330 (48.7)	247 (36.2)	249 (38.4)	445 (63.3)
4		1086 (40.0)	276 (40.7)	298 (43.6)	282 (43.5)	230 (32.7)
5		356 (13.1)	72 (10.6)	138 (20.2)	118 (18.2)	28 (4.0)
UACR, mg/gCr	2471	464 [106–1218]	1027 [334–2321]	685 [248–1249]	345 [86–874]	154 [34–467]
<300		1019 (41.2)	143 (23.4)	188 (29.9)	277 (46.4)	411 (64.8)
300–1000		718 (29.1)	157 (25.7)	226 (35.9)	194 (32.5)	141 (22.2)
>1000		734 (29.7)	311 (50.9)	215 (34.2)	126 (21.1)	82 (12.9)
Kidney disease	2713					
CGN		1204 (44.4)	274 (40.4)	332 (48.6)	288 (44.4)	310 (44.1)
DN		541 (19.9)	210 (31.0)	115 (16.8)	105 (16.2)	111 (15.8)
Nephrosclerosis		522 (19.2)	114 (16.8)	134 (19.6)	122 (18.8)	152 (21.6)
Others		446 (16.4)	80 (11.8)	102 (14.9)	134 (20.6)	130 (18.5)
Diabetes mellitus	2713	994 (36.6)	329 (48.5)	227 (33.2)	209 (32.2)	229 (32.6)
Smoking habits	2345					
Never smoker		1302 (55.5)	298 (51.7)	345 (58.2)	328 (58.2)	331 (54.1)
Active smoker		382 (16.3)	117 (20.3)	92 (15.5)	73 (12.9)	100 (16.3)
Ex-smoker		661 (28.2)	161 (28.0)	156 (26.3)	163 (28.9)	181 (29.6)
BMI, kg/m^2^	2453	23.5 (3.8)	24.0 (4.0)	23.4 (3.8)	23.0 (3.4)	23.6 (3.7)
Systolic blood pressure, mmHg	2682	131 (18)	136 (19)	132 (17)	130 (17)	127 (18)
Diastolic blood pressure, mmHg	2680	76 (12)	77 (12)	77 (12)	76 (11)	75 (12)
Total cholesterol, mg/dl	2281	194 (43)	198 (46)	195 (41)	192 (43)	191 (41)
History of any CVD	2713	647 (23.8)	194 (28.6)	136 (19.9)	158 (24.3)	159 (22.6)
ACEi/ARB	2713	2226 (82.0)	573 (84.5)	567 (83.0)	519 (80.0)	567 (80.7)
Frequency of eGFR measurement (times within the evaluation period)	2713	7 [5–10]	8 [6–11]	7 [5–10]	7 [5–10]	7 [5–10]

Data are expressed as N (%) for categorical values and mean (standard deviation) or median [interquartile range] for continuous values. Abbreviations: ARB, angiotensin receptor blocker; ACEi, angiotensin-converting enzyme inhibitor; BMI, body mass index; CGN, chronic glomerulonephritis; CKD, chronic kidney disease; CVD, cardiovascular disease; DN, diabetic nephropathy; eGFR, estimated glomerular filtration rate; UACR, urinary albumin–creatinine ratio.

### Slopes of estimated glomerular filtration rate

The mean eGFR slope over the 1-year evaluation estimated by a mixed-effects model was −1.80 ± 4.40 ml/min/1.73 m^2^ per year. The distributions of the slopes became narrower as the evaluation period became longer, regardless of the estimation method (Fig. S2).

### Association of estimated glomerular filtration rate slope with subsequent kidney failure with replacement therapy

The median follow-up periods were 5.1 [2.5 to 9.3], 5.0 [2.4 to 8.9], and 4.9 [2.1 to 8.0] years after the end of the 0.5-, 1-, and 2-year evaluation periods, respectively. In each evaluation period, 1051, 985, and 794 patients developed KFRT; 210, 201, and 171 died; and 444, 406, and 301 were lost to follow-up, respectively. The incidence rate of KFRT was 6.8–7.0 per 100 patient-years in the total population and increased with higher baseline CKD stage (Table 2). In adjusted Cox analysis, a slower eGFR decline was consistently associated with a lower risk of KFRT. The hazard ratios (HRs) of KFRT associated with a slower eGFR decline by 1 ml/min/1.73 m^2^ per year over 0.5, 1, and 2 years were 0.92 (0.90–0.93), 0.86 (0.85–0.87), and 0.66 (0.64–0.68), respectively. The magnitude of these associations was slightly decreased when UACR was added to the model, but remained largely consistent when UACR fold change was added (Table S3). Similar associations were obtained when the eGFR was calculated using least squares or when Fine–Gray models were used (Fig. S3).

**Table 2:** Association between eGFR slope (estimated using a mixed-effects model) and subsequent KFRT across different evaluation periods and CKD stages.

**Evaluation period**	**CKD stage**	***N***	**Subsequent KFRT**	**Incidence rate (/100 patients-year)**	**Unadjusted HR** ^a^ **(95% CI)**	**Adjusted HR** ^b^ **(95% CI)**
0.5 years	Total	2813	1051	6.8	0.93 (0.92–0.94)	0.92 (0.90–0.93)
	Stage 3	1281	206	2.3	0.93 (0.91–0.95)	0.94 (0.91–0.96)
	Stage 4	1111	525	9.4	0.90 (0.88–0.92)	0.90 (0.88–0.92)
	Stage 5	421	320	28.9	0.92 (0.89–0.95)	0.93 (0.90–0.96)
1 year	Total	2713	985	6.9	0.91 (0.90–0.92)	0.86 (0.85–0.87)
	Stage 3	1271	205	2.4	0.90 (0.88–0.92)	0.89 (0.87–0.91)
	Stage 4	1086	515	10.2	0.82 (0.80–0.85)	0.82 (0.80–0.84)
	Stage 5	356	265	29.6	0.71 (0.65–0.78)	0.81 (0.76–0.87)
2 year	Total	2336	794	7.0	0.78 (0.76–0.79)	0.66 (0.64–0.68)
	Stage 3	1171	190	2.8	0.70 (0.67–0.73)	0.66 (0.62–0.70)
	Stage 4	930	435	11.1	0.63 (0.60–0.67)	0.61 (0.58–0.65)
	Stage 5	235	169	31.2	0.44 (0.36–0.53)	0.38 (0.30–0.47)

a HR for the estimated deceleration in eGFR decline by 1 ml/min/1.73 m^2^ per year.

b Cox multivariate models were adjusted for age, sex, baseline eGFR, systolic blood pressure, CVD history, smoking status, and total cholesterol and stratified by research facility.

Abbreviations: CI, confidence interval; CKD, chronic kidney disease; eGFR, estimated glomerular filtration rate; HR, hazard ratio; KFRT, kidney failure with replacement therapy.

In an exploratory analysis, we examined whether the association between eGFR slope and subsequent KFRT changed after randomly reducing eGFR measurement frequency in patients with > 7 measurements/year (*N* = 1227 and 1045 for 1- and 2-year evaluation periods) (Table S4). The results were consistent regardless of the measurement frequency.

**Figure 2:**
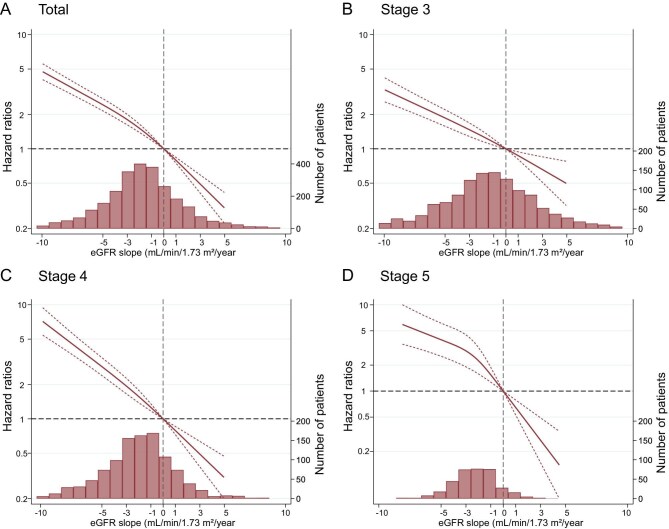
Associations between 1-year eGFR slope and subsequent KFRT in the total cohort and across CKD stages. Restricted cubic spline analysis revealed the association between eGFR slope and the risk of KFRT. The models were adjusted for age, sex, baseline eGFR, systolic blood pressure, CVD history, smoking status, and total cholesterol and stratified by research facility, in the total participants (A), stage 3 (B), stage 4 (C), and stage 5 (D). Abbreviations: CKD, chronic kidney disease; CVD, cardiovascular disease; eGFR, estimated glomerular filtration rate; KFRT, kidney failure with replacement therapy.

The restricted cubic splines showed a linear association between eGFR and subsequent KFRT in the total population (Fig. 2A) and in each CKD stage subgroup (Fig. 2B–D). The eGFR slope was consistently associated with KFRT, though the association weakened as the evaluation period shortened (Fig. S4).

### Stratified analysis

**Figure 3:**
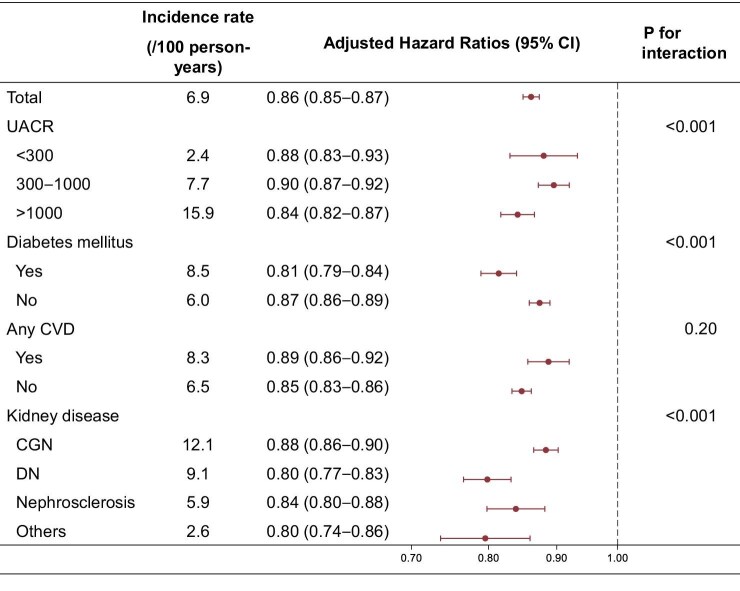
Hazard ratios (HRs) for KFRT associated with a 1 ml/min/1.73 m^2^ per year slower decline in eGFR slope over 1 year across subgroups. HR for KFRT associated with a 1 ml/min/1.73 m^2^ per year slower decline in eGFR slope, stratified by CKD stage, UACR category, history of DM, history of any CVD, and BMI. The models were adjusted for age, sex, baseline eGFR, systolic blood pressure, CVD history, smoking status, and total cholesterol and stratified by research facility. Abbreviations: BMI, body mass index; CGN, chronic glomerulonephritis; CKD, chronic kidney disease; CVD, cardiovascular disease; DM, diabetes mellitus; DN, diabetic nephropathy; KFRT, kidney failure with replacement therapy; UACR, urinary albumin–creatinine ratio.

Stratified analysis revealed that a slower eGFR decline was associated with a lower risk of KFRT in all subgroups examined. The differences in the magnitude of the association were small and not clinically meaningful (Fig. 3). Similar results were obtained in the stratified analysis with 0.5- and 2-year slope evaluation periods (Figs. S4 and S5).

### Estimation of clinically meaningful estimated glomerular filtration rate slope deceleration across different evaluation periods and chronic kidney disease stages

**Figure 4:**
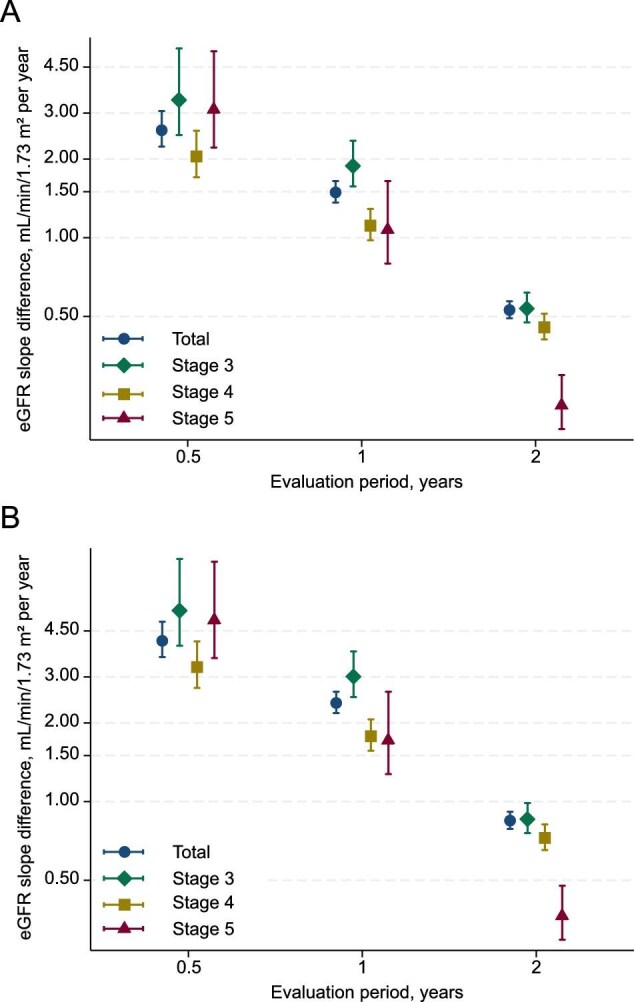
Estimated deceleration in eGFR decline associated with HRs of 0.8 and 0.7 for subsequent KFRT across CKD stages and slope evaluation periods. Deceleration in eGFR decline associated with an HR of 0.8 (A) and 0.7 (B) for subsequent KFRT were estimated across CKD stages and slope evaluation periods. The models were adjusted for the same covariates as in Table 2 and stratified by research facility. The models were adjusted for age, sex, baseline eGFR, systolic blood pressure, CVD history, smoking status, and total cholesterol and stratified by research facility. Abbreviations: CKD, chronic kidney disease; CVD, cardiovascular disease; eGFR, estimated glomerular filtration rate; KFRT, kidney failure with replacement therapy.

We then estimated the deceleration in eGFR decline associated with a 20% lower risk (HR of 0.8) of subsequent KFRT across different evaluation periods and CKD stages. In the total cohort, a slower eGFR decline by 2.58 (2.23–3.05), 1.49 (1.36–1.65), and 0.53 (0.49–0.57) ml/min/1.73 m^2^ per year over 0.5, 1, and 2 years, respectively, was associated with a 20% lower risk of subsequent KFRT (Fig. 4A). When eGFR slope was evaluated over 1 year, a slower eGFR decline by 1.88 (1.57–2.35), 1.11 (0.98–1.29), and 1.07 (0.80–1.65) ml/min/1.73 m^2^ per year was associated with an HR of 0.8 for subsequent KFRT in patients with CKD stages 3, 4, and 5, respectively. When we evaluated the eGFR slope over 2 years, the estimated eGFR decline associated with an HR of 0.8 for subsequent KFRT became smaller. Conversely, when we evaluated eGFR slope during the 0.5-year evaluation period, the estimated deceleration in eGFR decline associated with an HR of 0.8 for subsequent KFRT became far greater than that in the 1-year or 2-year eGFR slope analysis. The results of the slope of eGFR decline associated with a 30% lower risk are provided in Fig. 4B. Namely, a slower eGFR decline by 0.85 ml/min/1.73 m^2^ per year was associated with a 30% lower risk of subsequent KFRT. The estimated decelerations in eGFR decline are plotted against the HRs for subsequent KFRT in Fig. S6.

### Comparison between estimated glomerular filtration rate slope and percent decline in estimated glomerular filtration rate 

Finally, we compared the validity of eGFR slope and percent decline in eGFR as surrogate endpoints for KFRT. Overall, both surrogates were strongly associated with subsequent risk of KFRT, but the magnitude of the association varied across CKD stages and evaluation periods for each surrogate (Table 3). In particular, the effect of CKD stage on the association between each surrogate and KFRT was opposite for eGFR slope and percent decline in eGFR; the association between eGFR slope and KFRT was more pronounced with higher CKD stages, whereas the association between >30% and >40% decline in eGFR and KFRT was attenuated.

**Table 3:** Comparison of eGFR slope and >30% and >40% decline in eGFR in their association with KFRT.

		**Total**		**Stage 3**		**Stage 4**		**Stage 5**	
**Evaluation period**	**Surrogate endpoints** ^a^	***N***	**HR (95% CI)**	***N***	**HR (95% CI)**	***N***	**HR (95% CI)**	***N***	**HR (95% CI)**
1 year	eGFR slope, ml/min/1.73m^2^ per year								
	−5	2713	2.4 (2.2–2.7)	1271	1.9 (1.6–2.2)	1086	2.9 (2.4–3.4)	356	3.9 (2.5–6.1)
	−4		2.1 (1.9–2.4)		1.7 (1.4–1.9)		2.4 (2.0–2.8)		3.4 (2.2–5.3)
	−3		1.8 (1.6–2.0)		1.5 (1.3–1.7)		1.9 (1.7–2.7)		2.9 (1.9–4.5)
	−2		1.5 (1.4–1.7)		1.3 (1.2–1.4)		1.6 (1.4–1.8)		2.3 (1.6–3.2)
	−1		1.2 (1.2–1.3)		1.1 (1.1–1.2)		1.3 (1.2–1.4)		1.5 (1.3–1.9)
	0		Reference		Reference		Reference		Reference
	+1		0.79 (0.75–0.84)		0.87 (0.81–0.94)		0.79 (0.73–0.86)		0.64 (0.53–0.79)
	>30% decline in eGFR								
	Yes	418 (15%)	3.2 (2.7–3.7)	121 (10%)	3.6 (2.5–5.2)	205 (19%)	3.6 (2.9–4.5)	92 (26%)	2.5 (1.8–3.3)
	No	2295 (85%)	Reference	1150 (90%)	Reference	881 (81%)	Reference	264 (74%)	Reference
	>40% decline in eGFR								
	Yes	195 (7%)	3.8 (3.1–4.7)	52 (4%)	4.7 (2.8–7.9)	104 (10%)	4.1 (3.1–5.4)	39 (11%)	3.8 (2.4–5.9)
	No	2518 (93%)	Reference	1219 (96%)	Reference	982 (90%)	Reference	317 (89%)	Reference
2 years	eGFR slope, ml/min/1.73m^2^ per year								
	−5	2336	10 (8.1–12)	1171	8.8 (6.2–13)	930	12 (8.9–16)	235	181 (55–592)
	−4		6.9 (5.6–8.4)		5.9 (4.2–8.4)		7.5 (5.7–9.8)		53 (22–127)
	−3		4.6 (3.7–5.6)		4.0 (2.9–5.5)		4.6 (3.6–6.0)		15 (7.7–30)
	−2		3.0 (2.5–3.5)		2.6 (2.0–3.4)		2.8 (2.3–3.5)		4.7 (2.5–9.0)
	−1		1.8 (1.6–2.0)		1.6 (1.4–1.9)		1.7 (1.5–2.0)		1.9 (1.2–3.0)
	0		Reference		Reference		Reference		Reference
	+1		0.54 (0.48–0.62)		0.60 (0.49–0.74)		0.59 (0.50–0.69)		0.54 (0.33–0.87)
	>30% decline in eGFR								
	Yes	650 (28%)	4.3 (3.6–5.0)	230 (20%)	6.6 (4.8–9.2)	309 (33%)	4.1 (3.3–5.1)	111 (47%)	2.9 (2.0–4.2)
	No	1686 (72%)	Reference	941 (80%)	Reference	621 (67%)	Reference	124 (53%)	Reference
	>40% decline in eGFR								
	Yes	375 (16%)	4.5 (3.8–5.3)	119 (10%)	6.6 (4.6–9.6)	191 (21%)	4.7 (3.7–5.8)	65 (28%)	3.8 (2.5–5.8)
	No	1961 (84%)	Reference	1052 (90%)	Reference	739 (79%)	Reference	170 (72%)	Reference

^a^eGFR slopes were obtained using a mixed-effects model, and >30% or >40% decline in eGFR was defined as occurrence of more than 30 or 40% drop of eGFR from the baseline at any one time during the evaluation period. The percent decline in eGFR was defined by the following equation: (last eGFR at evaluation period – first eGFR)/(first eGFR)×100.

HRs and CIs were obtained from restricted cubic splines for each evaluation period. Cox multivariate models were adjusted for the same covariates as shown in Table 2 and stratified by research facility. Abbreviations: CI, confidence interval; eGFR, estimated glomerular filtration rate; HR, hazard ratio; KFRT, kidney failure with replacement therapy.

## DISCUSSION

In this prospective cohort of 2966 Japanese patients with CKD stages 3–5 who were enrolled in the CKD-JAC Study, we confirmed eGFR slope as a strong predictor of KFRT, supporting its potential use as a surrogate endpoint. Our results extend the findings of the CKD-PC study, which included primarily Western cohorts, to Asian CKD populations and support the generalizability of the validity of eGFR slope across different races and regions. Furthermore, we found the strong association between eGFR slope and subsequent KFRT risk not only in individuals with CKD stage 3 but also in those with CKD stages 4 and 5. The overall association between eGFR slope and KFRT risk was less pronounced when eGFR slope was evaluated over shorter intervals, but eGFR slope still appeared to be useful for predicting KFRT risk when the analysis was limited to patients with CKD stages 4 and 5. These findings provide valuable insights for the future design of clinical trials in CKD patients, especially those with more advanced CKD.

In the previous analysis of the CKD-PC, a slower decline iin eGFR by 0.75 ml/min/1.73 m^2^ per year over 2 years was associated with a 30% lower risk of subsequent KFRT. In the present analysis of the CKD-JAC Study, when we estimated eGFR slope over 2 years, a slower eGFR decline by 0.85 ml/min/1.73 m^2^ per year was associated with a 30% lower risk of subsequent KFRT. These findings suggest that the associations between eGFR slope and KFRT risk are similar in magnitude in participants in the CKD-PC and those in the CKD-JAC Study, supporting the potential of eGFR slope as a surrogate endpoint in different racial populations.

The validity of the eGFR slope as a surrogate endpoint has been investigated primarily in patients with mild-to-moderate CKD (eGFR > 30 ml/min/1.73 m^2^) [5, 6]. However, an increasing number of clinical trials have recently included patients with CKD stage 4 or 5 [7, 9, 10, 18]. In a subanalysis of dapagliflozin use in patients with stage 4 CKD, there was a significant difference in eGFR slope, although no significant differences were observed in hard outcomes [7]. This underscores the utility of the eGFR slope for increasing statistical power, even in populations with advanced CKD, and highlights its potential for efficient trial design by minimizing the observation period and the number of participants.

For efficient clinical trial design, it is also important to consider the incidence of surrogate endpoints in the study population. Given the low incidence of KFRT within 2 years in patients with stage 4 CKD, the use of eGFR slope instead of KFRT may be a reasonable approach. In contrast, for patients with stage 5 CKD, of whom approximately 40% reached KFRT within 2 years in the CKD-JAC Study, the use of KFRT alone or a composite of KFRT and a 30% decline in eGFR would be more feasible than eGFR slope. However, if clinical trials are to be designed with a 1-year observation period, the composite of KFRT and 30% decline in eGFR will occur with a lower incidence, and eGFR slope may still serve as a reasonable endpoint. Regardless of CKD stage, the association between eGFR and KFRT decreased with shorter evaluation periods. However, it is noteworthy that the association between eGFR slope and subsequent KFRT was pronounced in patients with later CKD stages, whereas the association was attenuated for >30% or >40% decline in eGFR. This may further support the potential advantage of the eGFR slope over >30% or >40% decline in eGFR in this setting. Further research using data from randomized clinical trials is needed to determine the superiority of eGFR slope and percent decline in eGFR across CKD stages.

The validity of using the eGFR slope as a surrogate for advanced CKD may be influenced by regional variations in eGFR slope and the eGFR at KRT initiation. In Japan and Taiwan, the average eGFR at KRT initiation is approximately 5–6 ml/min/1.73 m^2^, whereas in the United States, it exceeds 10 ml/min/1.73 m^2^ [12, 19], likely due to a higher incidence of congestive heart failure. Similarly, in Europe and Canada, KRT tends to be initiated at relatively higher eGFR [12]. These variations impose limitations on the external validity of our study. However, with recent dramatic advancements in heart failure treatment [20, 21], there may be a shift toward initiating KRT at a lower eGFR in the future. If such a shift occurs, the significance of this study investigating the utility of the eGFR slope in advanced CKD could extend beyond Japan and provide international value.

Recently, there has been growing interest in using changes in UACR as a novel surrogate endpoint [2, 6, 22–24]. However, the validity of the surrogacy of UACR change is limited in patients with low albuminuria. We demonstrated that even in such patients, eGFR slope serves as a valid surrogate endpoint. Furthermore, the association between eGFR slope and KFRT persisted after adjustment for changes in UACR over the same evaluation period. Taken together, our results support the hierarchy of endpoints for kidney disease progression, with eGFR slope superior to UACR. Further studies are needed to investigate whether the combination of eGFR slope and UACR change can serve as a more reliable surrogate for KFRT.

Several limitations should be acknowledged. First, this observational study did not examine changes after specific treatment interventions, nor did it compare eGFR slopes between different treatment arms. In addition, this study did not account for different treatment responses or acute and chronic effects of treatment. Therefore, we should be cautious about translating these results into clinical trials. Second, in addition to the limited sample size, this study included only Japanese CKD patients, and the majority of participants received care from board-certified nephrologists, which may limit the generalizability of the results. Third, potential selection bias may have occurred by restricting the analysis to patients with eGFR measurements every 6 months and excluding those censored during the evaluation period. Another potential bias was eGFR measurement frequency, which may have been influenced by clinical circumstances; however, the results were largely consistent across evaluation periods regardless of the measurement frequency.

In conclusion, we demonstrated a strong association between eGFR slope and the risk of KFRT across CKD stages in Japanese CKD patients. Our results extrapolate the surrogacy of eGFR slope, not only to Asian CKD patients but also to those with more advanced CKD. Our results also demonstrate that the evaluation period of eGFR slope and CKD stage are important determinants of clinically meaningful differences in eGFR slope, and suggest that a shorter evaluation period than 2 years may be feasible for patients with late-stage CKD.

## Supplementary Material

sfae398_Supplemental_File

## Data Availability

Owing to personal information protection laws, our data are not freely available. The data underlying this article will be shared upon reasonable request to the corresponding author.
