# Supplementary material for: Clinically meaningful eGFR slope as a surrogate endpoint differs across CKD stages and slope evaluation periods: the CKD-JAC study
Source: Clin Kidney J. 2025 Jan 13;18(2):sfae398. doi: 10.1093/ckj/sfae398 (PMC11822292; doi:10.1093/ckj/sfae398)
Supplement: sfae398_Supplemental_File [file sfae398_supplemental_file.docx]

**Clinically Meaningful eGFR Slope as a Surrogate Endpoint Differs across CKD Stages and Slope Evaluation Periods: The CKD-JAC Study**

Short title: eGFR slope as a surrogate endpoint

**-Supplementary material-**

[**Supplementary Methods 2**](#_Toc179709762)

[**Supplementary References 4**](#_Toc179709763)

[**Table S1.** Baseline characteristics of the whole cohort **5**](#_Toc179709764)

[**Table S2.** Number of events and event-free survival in the whole cohort and across CKD stages **6**](#_Toc179709765)

[**Table S3.** Comparison of the association between eGFR slope and KFRT with and without adjustment for UACR and its fold change during 2 years **7**](#_Toc179709766)

[**Table S4.** Comparison of the association between eGFR slope and KFRT by reducing the frequency of eGFR measurements in patients with frequent measurement of eGFR **8**](#_Toc179709767)

[**Figure S1.** Cumulative incidence of kidney events across CKD stages **9**](#_Toc179709768)

[**Figure S2.** Distributions of eGFR slopes estimated using mixed effects models and least squares **10**](#_Toc179709769)

[**Figure S3.** Hazard ratios for KFRT associated with a 1 mL/min/1.73 m^2^ per year slower decline in eGFR slope estimated over different evaluation periods using mixed effects models and least squares **11**](#_Toc179709770)

[**Figure S4.** Associations eGFR slope and subsequent KFRT across different evaluation periods **12**](#_Toc179709771)

[**Figure S5.** Hazard ratios for KFRT associated with a 1 mL/min/1.73 m^2^ per year slower decline in eGFR slope over 0.5 years across subgroups **13**](#_Toc179709772)

[**Figure S6.** Hazard ratios for KFRT associated with a 1 mL/min/1.73 m^2^ per year slower decline in eGFR slope over 2 years across subgroups **14**](#_Toc179709773)

[**Figure S7.** Estimated hazard ratios for KFRT associated with slower decline in eGFR slope across CKD stages and slope evaluation periods **15**](#_Toc179709774)

Supplementary Methods

***Covariates***

Clinical data were collected at enrollment, and laboratory parameters of blood and urine samples were measured centrally using standardized assays, as previously reported (S1).

Biometric data, such as body mass index (BMI) and blood pressure, were collected using standardized procedures. Diabetes mellitus (DM) was defined as a fasting blood glucose ≥126 mg/dL, a random blood glucose ≥200 mg/dL, or the use of antidiabetic drugs. A history of CVD consisted of coronary artery disease, congestive heart failure, peripheral artery disease, and stroke (S2). The causes of kidney disease included chronic glomerulonephritis, diabetic kidney disease, nephrosclerosis, and others based on medical records. Missing data for covariates were <5% for systolic and diastolic blood pressure, <10% for BMI and urinary albumin-creatinine ratio (UACR), and 14.6% for smoking status.

***Definition of a 30 or 40% decline in eGFR***

We defined “a 30% decline in eGFR” as occurring when a 30% decline from baseline was observed at three consecutive visits, with the occurrence defined at the time of the first 30% decline. If KFRT occurred after a 30% decline in eGFR with no available second or third measurement, we defined that the patient achieved this endpoint at the first time of that 30% decline. A 40% decline was defined similarly.

***Additional analyses***

We performed stratified analysis by CKD stage, UACR category, DM, history of CVD, BMI, and cause of kidney disease. We also used restricted cubic spline (RCS) analysis to examine potential nonlinear associations between eGFR slope and the incidence of KFRT with 3 knots (10th, 50th, and 90th percentiles) (S3).

We considered a clinically meaningful risk reduction to be in the range between 0.7 and 0.8 of the hazard ratio (HR) for KFRT (S4). This notion is consistent with prior clinical trials showing a risk reduction of 28% for losartan (S5) and 18% for finerenone (S6) as proof of efficacy. We estimated the degree of decline in eGFR slope associated with a HR of 0.8 for KFRT in each evaluation period and across CKD stages. To determine the difference in the eGFR slope that would result in an HR of 0.7 or 0.8, we calculated the corresponding values based on a logarithmic transformation of the HR per unit increase in the explanatory variable, using 0.7 and 0.8 as the base.

We performed an additional exploratory analysis in patients whose eGFR measurement frequency was greater than the median and compared the association between the eGFR slope and KFRT after randomly reducing the frequency of eGFR measurement to once every 3, 4, or 6 months.

In addition, we included UACR and its fold change over 2 years as covariates in multivariable models. In the CKD-JAC study, UACR was measured at baseline and 2 years after enrollment, so this analysis was restricted to patients for whom both baseline and 2-year values were available (n=1751). Changes in albuminuria were examined on a logarithmic scale with a base of 2 to allow interpretation as a fold change. Finally, we compared the associations of eGFR slope and >30% or >40% decline in eGFR with the risk of KFRT.

Multiple imputations with chained equation was used to handle missing data (S7). All the statistical tests were two-sided. P <0.05 indicated statistical significance. All analyses were performed using Stata 18.0 (StataCorp, TX, USA).

Supplementary References

S1. Imai E, Matsuo S, Makino H et al. Chronic Kidney Disease Japan Cohort (CKD-JAC) study: design and methods. Hypertension research 2008; 31: 1101–1107.

S2. Imaizumi T, Hamano T, Fujii N et al. Cardiovascular disease history and β-blocker prescription patterns among Japanese and American patients with CKD: a cross-sectional study of the CRIC and CKD-JAC studies. Hypertension Research 2021; 44: 700–710.

S3. Desquilbet L, Mariotti F. Dose-response analyses using restricted cubic spline functions in public health research. Statistics in Medicine 2010; 29: 1037–1057.

S4. Levey AS, Gansevoort RT, Coresh J et al. Change in Albuminuria and GFR as End Points for Clinical Trials in Early Stages of CKD: A Scientific Workshop Sponsored by the National Kidney Foundation in Collaboration With the US Food and Drug Administration and European Medicines Agency [Internet]. In: American Journal of Kidney Diseases. Am J Kidney Dis, 2020 [cited 2022 Aug 3] ; 84–104Available from: https://pubmed.ncbi.nlm.nih.gov/31473020/

S5. Brenner BM, Cooper ME, de Zeeuw D et al. Effects of Losartan on Renal and Cardiovascular Outcomes in Patients with Type 2 Diabetes and Nephropathy. New England Journal of Medicine *[Internet]* 2001; [cited 2022 Oct 8] 345: 861–869. Available from: https://pubmed.ncbi.nlm.nih.gov/11565518/

S6. Bakris GL, Agarwal R, Anker SD et al. Effect of Finerenone on Chronic Kidney Disease Outcomes in Type 2 Diabetes. New England Journal of Medicine 2020; 383: 2219–2229.

S7. White IR, Royston P, Wood AM. Multiple imputation using chained equations: Issues and guidance for practice. Statistics in Medicine 2011; 30: 377–399.

Table S1. Baseline characteristics of the whole cohort

|  | Total  (2,966) | Stage 3  (1,353) | Stage 4  (1148) | Stage 5  (465) |
| --- | --- | --- | --- | --- |
| Age, years | 60 (12) | 59 (12) | 61 (11) | 62 (11) |
| Male | 1,841 (62.1) | 869 (64.2) | 705 (61.4) | 267 (57.4) |
| eGFR, ml/min/1.73m^2^ | 29 (12) | 40 (7) | 23 (4) | 12 (2) |
| UACR, mg/gCr | 492  (115-1314) | 245  (52-813) | 653  (196-1427) | 1085  (454-2026) |
| <300 | 1,074 (40.1) | 650 (53.8) | 347 (33.2) | 77 (18.1) |
| 300-1000 | 757 (28.3) | 311 (25.7) | 319 (30.5) | 127 (29.9) |
| >1000 | 848 (31.7) | 247 (20.4) | 380 (36.3) | 221 (52.0) |
| Kidney disease |  |  |  |  |
| CGN | 1,285 (43.3) | 634 (46.9) | 471 (41.0) | 180 (38.7) |
| DN | 624 (21.0) | 245 (18.1) | 245 (21.3) | 134 (28.8) |
| Nephrosclerosis | 567 (19.1) | 226 (16.7) | 256 (22.3) | 85 (18.3) |
| Others | 490 (16.5) | 248 (18.3) | 176 (15.3) | 66 (14.2) |
| Diabetes mellitus | 1,117 (37.7) | 477 (35.3) | 444 (38.7) | 196 (42.2) |
| Smoking habits |  |  |  |  |
| Never smoker | 1,390 (55.1) | 628 (54.5) | 535 (54.7) | 227 (58.2) |
| Active smoker | 425 (16.9) | 203 (17.6) | 154 (15.7) | 68 (17.4) |
| Ex-smoker | 706 (28.0) | 322 (27.9) | 289 (29.6) | 95 (24.4) |
| BMI, kg/m2 | 23.5 (3.8) | 23.8 (3.8) | 23.3 (3.8) | 23.2 (3.7) |
| Systolic Blood Pressure, mmHg | 132 (19) | 130 (18) | 132 (19) | 136 (19) |
| Diastolic Blood Pressure, mmHg | 76 (12) | 76 (11) | 76 (12) | 76 (12) |
| Total cholesterol, mg/dL | 194 (43) | 198 (44) | 192 (41) | 187 (46) |
| History of any CVD | 726 (24.5) | 291 (21.5) | 296 (25.8) | 139 (29.9) |
| ACEi/ARB | 2,427 (81.8) | 1,079 (79.7) | 967 (84.2) | 381 (81.9) |

Data are expressed as N (%) for categorical values and mean (standard deviation) or median [interquartile range] for continuous values. Abbreviations: eGFR, estimated glomerular filtration rate; UACR, urinary albumin: creatinine ratio; CGN, chronic glomerulonephritis; DN, diabetic nephropathy; BMI, body mass index; CVD, cardiovascular disease; ACEi, angiotensin-converting enzyme inhibitor; ARB, angiotensin receptor blocker.

**Table S2. Number of events and event-free survival in the whole cohort and across CKD stages**

| **CKD stage** | **Number of events^*^** | **Incidence rate (/100 patient-years)** | **Median event-free survival (year)** | **Cumulative number of events**  **within the periods (year)** | | |
| --- | --- | --- | --- | --- | --- | --- |
|  |  |  |  | **0–0.5** | **0–1** | **0–2** |
| **Event 1: Composite of 30% decline in eGFR and KFRT** | | | | | | |
| Total  (2966) | 1871 | 14.5 (13.9–15.2) | 4.4 | 118 | 333 | 739 |
| Stage 3  (1353) | 638 | 8.3 (7.7–9.0) | 8.3 | 16 | 47 | 141 |
| Stage 4  (1148) | 837 | 19.7 (18.4–21.1) | 3.5 | 45 | 143 | 326 |
| Stage 5  (465) | 396 | 41.7 (37.8–46.1) | 1.5 | 57 | 143 | 272 |
| **Event 2: Composite of 40% decline in eGFR and KFRT** | | | | | | |
| Total  (2966) | 1642 | 11.4 (10.8–11.9) | 6.0 | 72 | 214 | 541 |
| Stage 3  (1353) | 512 | 6.0 (5.5–6.6) | 10.2 | 6 | 20 | 79 |
| Stage 4  (1148) | 751 | 15.5 (14.4–16.7) | 4.4 | 24 | 77 | 233 |
| Stage 5  (465) | 379 | 34.7 (31.4–38.4) | 1.9 | 42 | 117 | 229 |
| **Event 3: KFRT** | | | | | | |
| Total  (2966) | 1097 | 6.4 (6.0–6.8) | 10.4 | 31 | 106 | 273 |
| Stage 3  (1353) | 212 | 2.2 (1.9–2.5) | - | 0 | 3 | 10 |
| Stage 4  (1148) | 535 | 8.8 (8.1–9.6) | 7.2 | 5 | 18 | 90 |
| Stage 5  (465) | 350 | 26.8 (24.2–29.8) | 2.6 | 26 | 85 | 173 |

*Followed up until the end of the study.

Abbreviations: CKD, chronic kidney disease; eGFR, estimated glomerular filtration rate; KFRT, kidney failure with replacement therapy.

Table S3. Comparison of the association between eGFR slope and KFRT with and without adjustment for UACR and its fold change during 2 years

| CKD stage | Model | HR | (95% CI) | P value |
| --- | --- | --- | --- | --- |
| Total  (n = 2,336) | Mixed-effects |  |  |  |
|  | Multivariable adjustment | 0.65 | (0.63–0.68) | <0.001 |
|  | + UACR | 0.69 | (0.66–0.72) | <0.001 |
|  | + UACR change | 0.69 | (0.66–0.72) | <0.001 |
|  | Least-squares |  |  | <0.001 |
|  | Multivariable adjustment | 0.70 | (0.68–0.72) | <0.001 |
|  | + UACR | 0.73 | (0.71–0.76) | <0.001 |
|  | + UACR change | 0.73 | (0.71–0.76) | <0.001 |
| Stage 3  (n = 1,171) | Mixed-effects |  |  |  |
|  | Multivariable adjustment | 0.64 | (0.60–0.69) | <0.001 |
|  | + UACR | 0.68 | (0.63–0.74) | <0.001 |
|  | + UACR change | 0.69 | (0.64–0.75) | <0.001 |
|  | Least-squares |  |  | <0.001 |
|  | Multivariable adjustment | 0.69 | (0.65–0.73) | <0.001 |
|  | + UACR | 0.73 | (0.68–0.77) | <0.001 |
|  | + UACR change | 0.73 | (0.69–0.78) | <0.001 |
| Stage 4  (n = 930) | Mixed-effects |  |  |  |
|  | Multivariable adjustment | 0.60 | (0.56–0.64) | <0.001 |
|  | + UACR | 0.63 | (0.59–0.68) | <0.001 |
|  | + UACR change | 0.65 | (0.60–0.69) | <0.001 |
|  | Least-squares |  |  | <0.001 |
|  | Multivariable adjustment | 0.65 | (0.61–0.69) | <0.001 |
|  | + UACR | 0.68 | (0.64–0.72) | <0.001 |
|  | + UACR change | 0.69 | (0.65–0.73) | <0.001 |
| Stage 5  (n = 235) | Mixed-effects |  |  |  |
|  | Multivariable adjustment | 0.31 | (0.23–0.42) | <0.001 |
|  | + UACR | 0.32 | (0.24–0.44) | <0.001 |
|  | + UACR change | 0.33 | (0.24–0.45) | <0.001 |
|  | Least-squares |  |  | <0.001 |
|  | Multivariable adjustment | 0.38 | (0.29–0.48) | <0.001 |
|  | + UACR | 0.39 | (0.30–0.51) | <0.001 |
|  | + UACR change | 0.40 | (0.30–0.52) | <0.001 |

The association between eGFR slope and KFRT did not change when albuminuria and its two-year fold change were added to the multivariate model. UACR, urinary albumin-creatinine ratio

Table S4. Comparison of the association between eGFR slope and KFRT by reducing the frequency of eGFR measurements in patients with frequent measurement of eGFR

| Evaluation period | Frequency of eGFR measurement | Slope estimation | HR | 95% CI |
| --- | --- | --- | --- | --- |
| 1 year | >7 times/year (not reduced) | Mixed-effects | 0.87 | (0.85–0.89) |
| (N = 1,227) |  | Least-squares | 0.90 | (0.89–0.92) |
|  | 4 times/year | Mixed-effects | 0.85 | (0.83–0.88) |
|  |  | Least-squares | 0.93 | (0.91–0.94) |
|  | 3 times/year | Mixed-effects | 0.83 | (0.81–0.86) |
|  |  | Least-squares | 0.94 | (0.92–0.95) |
|  | 2 times/year | Mixed-effects | 0.81 | (0.78–0.84) |
|  |  | Least-squares | 0.93 | (0.92–0.95) |
| 2 years | >7 times/year (not reduced) | Mixed-effects | 0.64 | (0.61–0.67) |
| (N = 1,045) |  | Least-squares | 0.68 | (0.65–0.71) |
|  | 4 times/year | Mixed-effects | 0.65 | (0.62–0.68) |
|  |  | Least-squares | 0.72 | (0.70–0.75) |
|  | 3 times/year | Mixed-effects | 0.63 | (0.60–0.67) |
|  |  | Least-squares | 0.73 | (0.71–0.76) |
|  | 2 times/year | Mixed-effects | 0.61 | (0.58–0.65) |
|  |  | Least-squares | 0.78 | (0.75–0.80) |

GFR, glomerular filtration rate; KFRT, kidney failure replacement therapy; HR, hazard ratio; CI, confidence interval


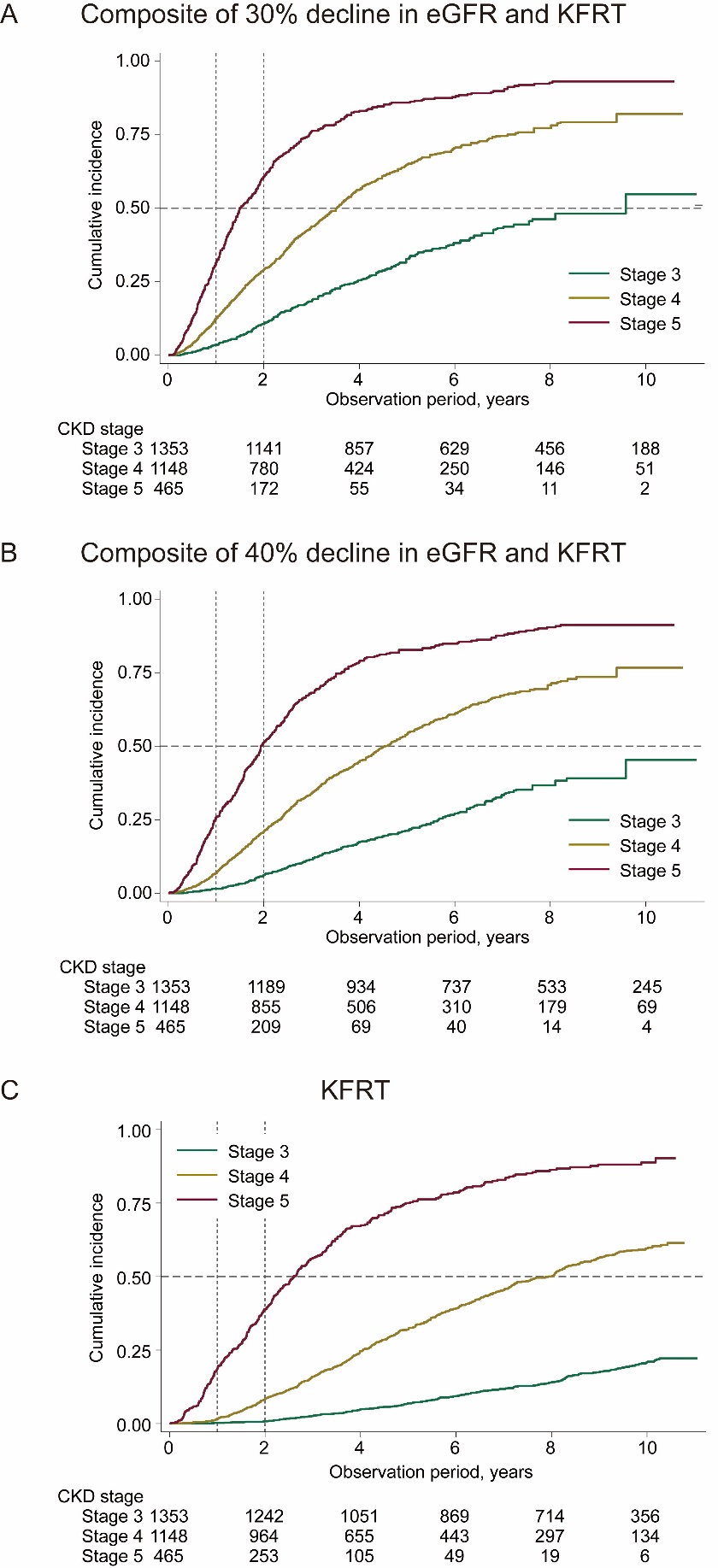


Figure S1. Cumulative incidence of kidney events across CKD stages

**A.** Composite of 30% decline in eGFR and KFRT**. B.** Composite of 40% decline in eGFR and KFRT**. C.** KFRT alone**.** Analysis was conducted using the whole cohort of the CKD-JAC, stratified by CKD stage. Abbreviations: eGFR, estimated glomerular filtration rate; KFRT, kidney failure with replacement therapy; CKD, chronic kidney disease.


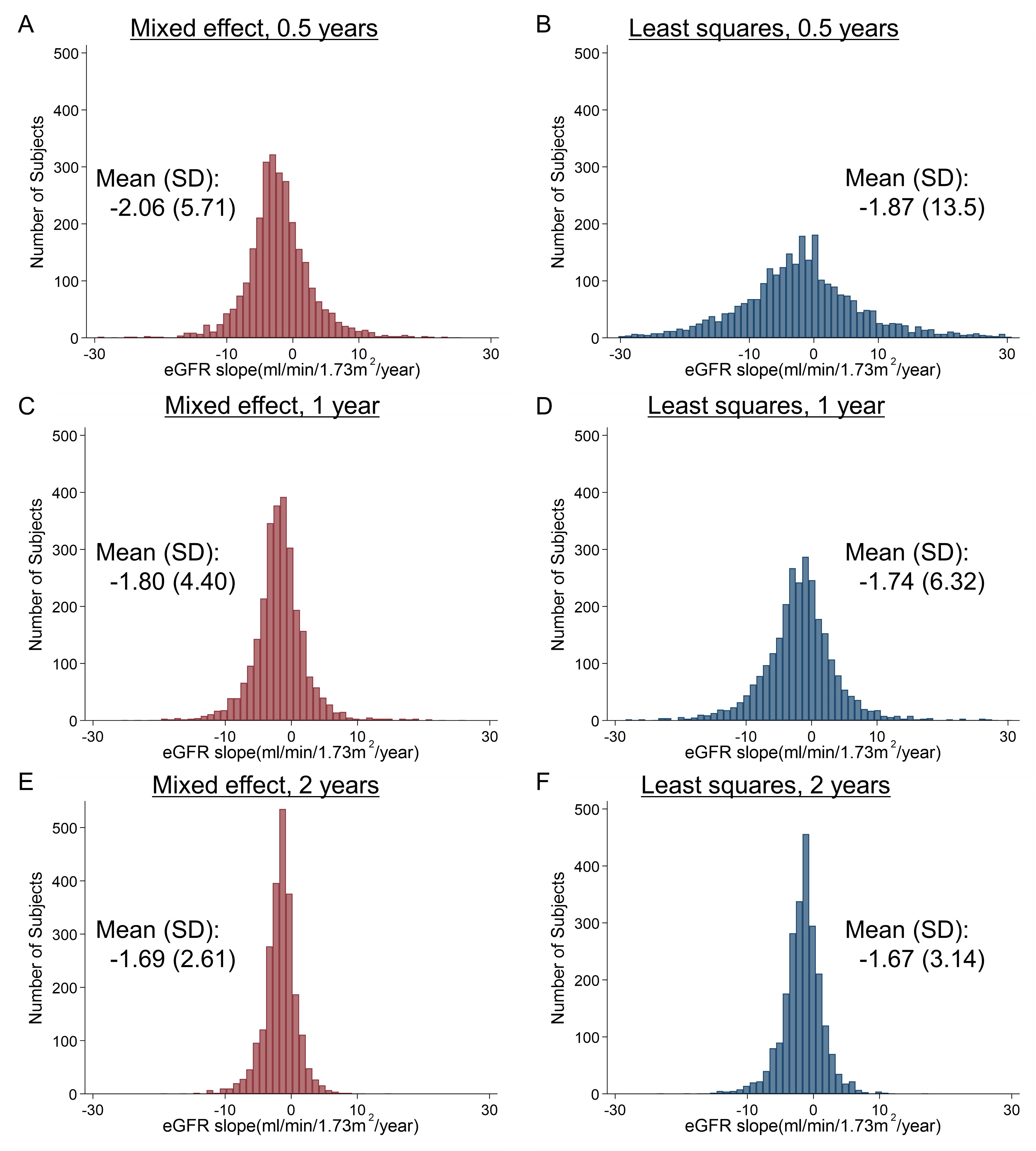


Figure S2. Distributions of eGFR slopes estimated using mixed effects models and least squares

eGFR slopes were estimated using mixed-effects models (**A, C, E**) and least squares (**B, D, F**). Slope evaluation periods were 0.5 years (**A, B**), 1 year (**C, D**), and 2 years (**E, F**)**.** Median [IQR] times of eGFR measurements were 4 [3 to 5], 7 [5 to 9], and 14 [11 to 19] for 0.5-, 1-, and 2-year evaluation periods, respectively. Abbreviations: eGFR, estimated glomerular filtration rate; SD, standard deviation

Figure S3. Hazard ratios for KFRT associated with a 1 mL/min/1.73 m^2^ per year slower decline in eGFR slope estimated over different evaluation periods using mixed effects models and least squares


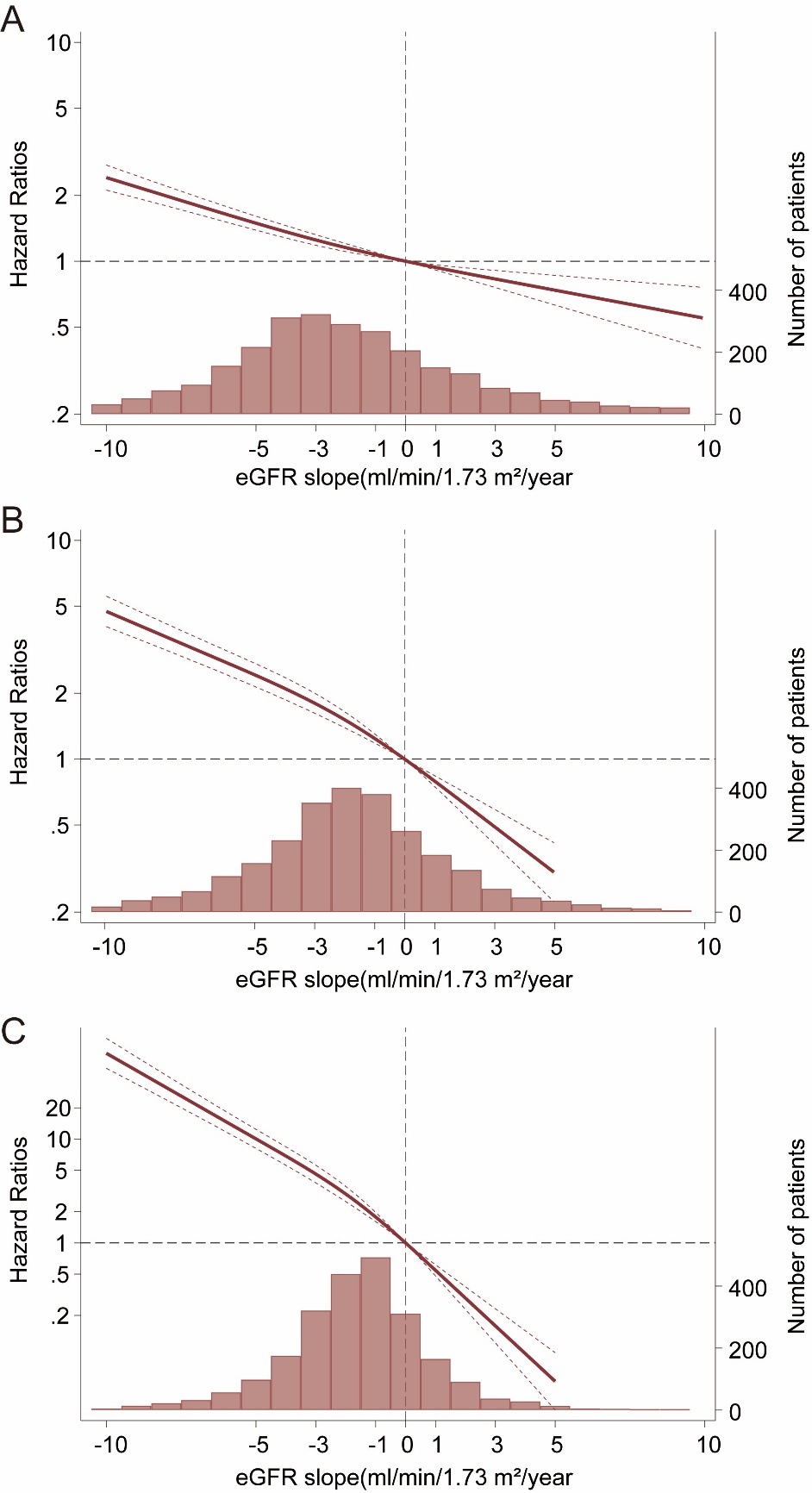


Figure S4. Associations eGFR slope and subsequent KFRT across different evaluation periods

Restricted cubic spline analysis revealed the association between eGFR slope and the risk of KFRT using different evaluation periods: 0.5 years (**A**); 1 year (**B**); and 2 years (**C**). The models were adjusted for age, sex, baseline eGFR, systolic blood pressure, CVD history, smoking status, and total cholesterol and stratified by research facility. Abbreviations: eGFR, estimated glomerular filtration rate; KFRT, kidney failure with replacement therapy.


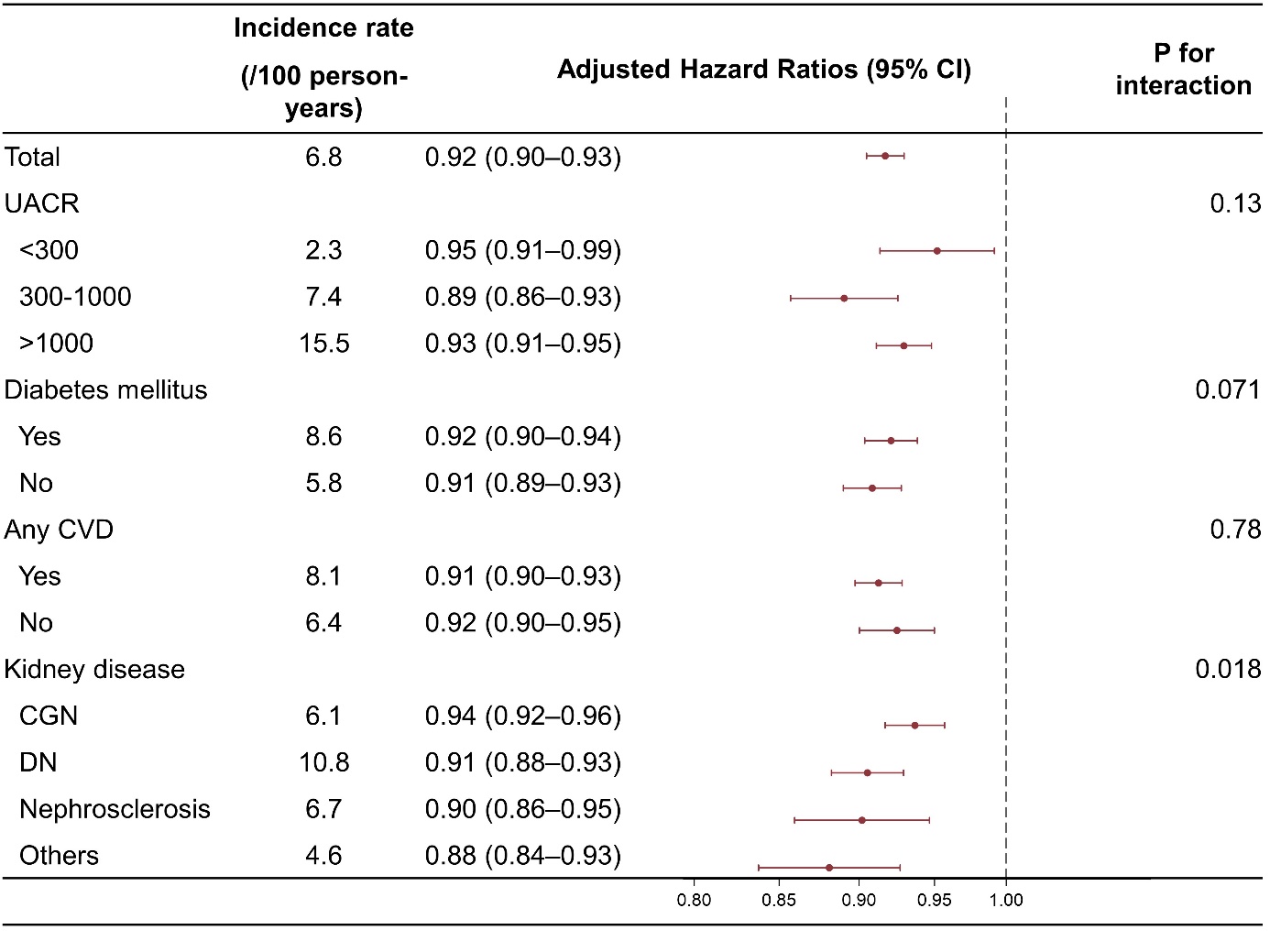


Figure S5. Hazard ratios for KFRT associated with a 1 mL/min/1.73 m^2^ per year slower decline in eGFR slope over 0.5 years across subgroups

Analysis was performed with stratification by CKD stage, UACR category, history of DM, history of any CVD, and BMI. Models were adjusted for age, sex, baseline eGFR, systolic blood pressure, CVD history, smoking status, and total cholesterol and stratified by research facility. Abbreviations: KFRT, kidney failure with replacement therapy; CKD, chronic kidney disease; UACR, urinary albumin: creatinine ratio; DN, diabetic nephropathy; CVD, cardiovascular disease; CGN, chronic glomerulonephritis.


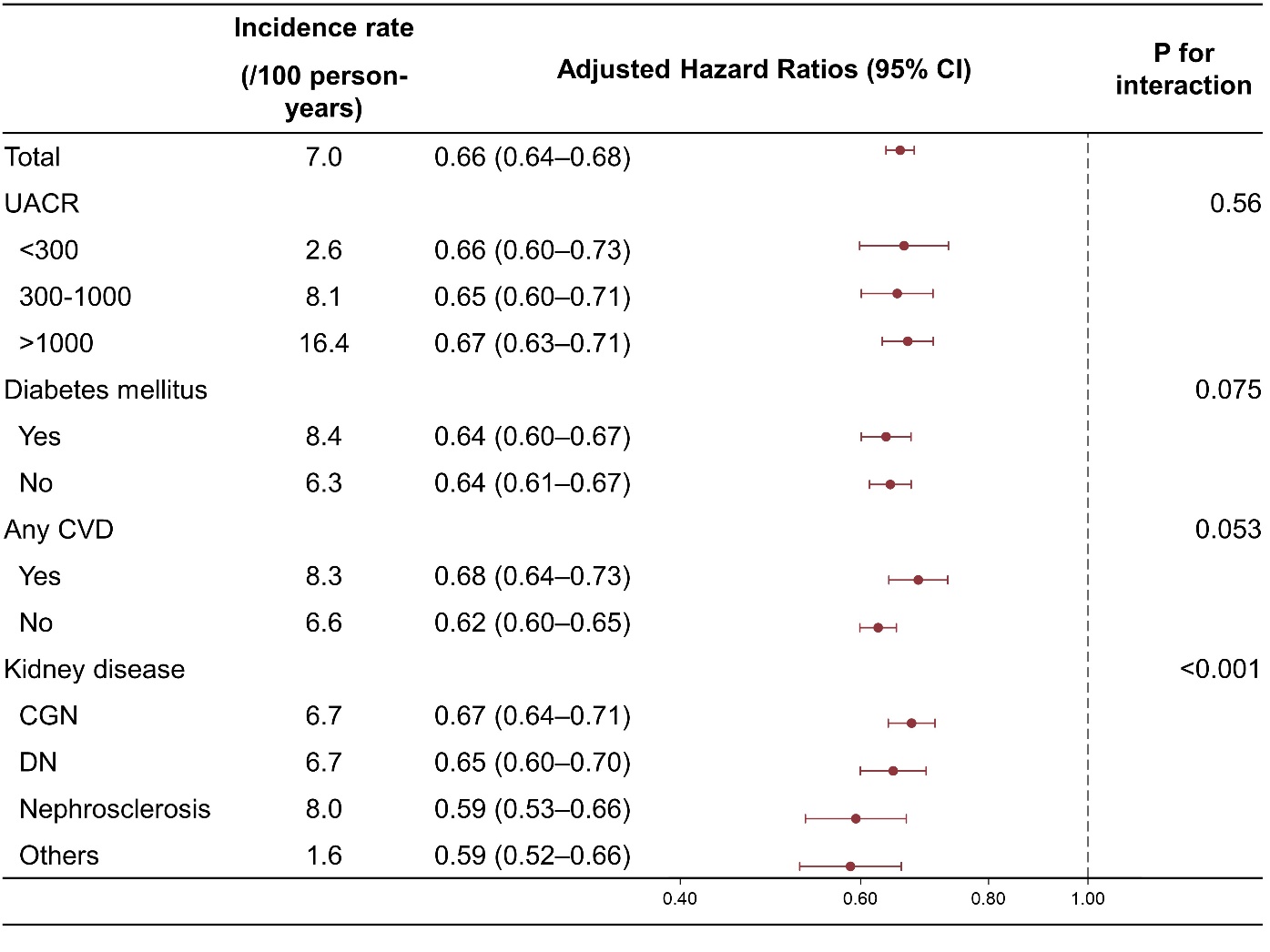


Figure S6. Hazard ratios for KFRT associated with a 1 mL/min/1.73 m^2^ per year slower decline in eGFR slope over 2 years across subgroups

Analysis was performed with stratification by CKD stage, UACR category, history of DM, history of any CVD, and BMI. Models were adjusted for age, sex, baseline eGFR, systolic blood pressure, CVD history, smoking status, and total cholesterol and stratified by research facility. Abbreviations: KFRT, kidney failure with replacement therapy; CKD, chronic kidney disease; UACR, urinary albumin: creatinine ratio; DN, diabetic nephropathy; CVD, cardiovascular disease; CGN, chronic glomerulonephritis.


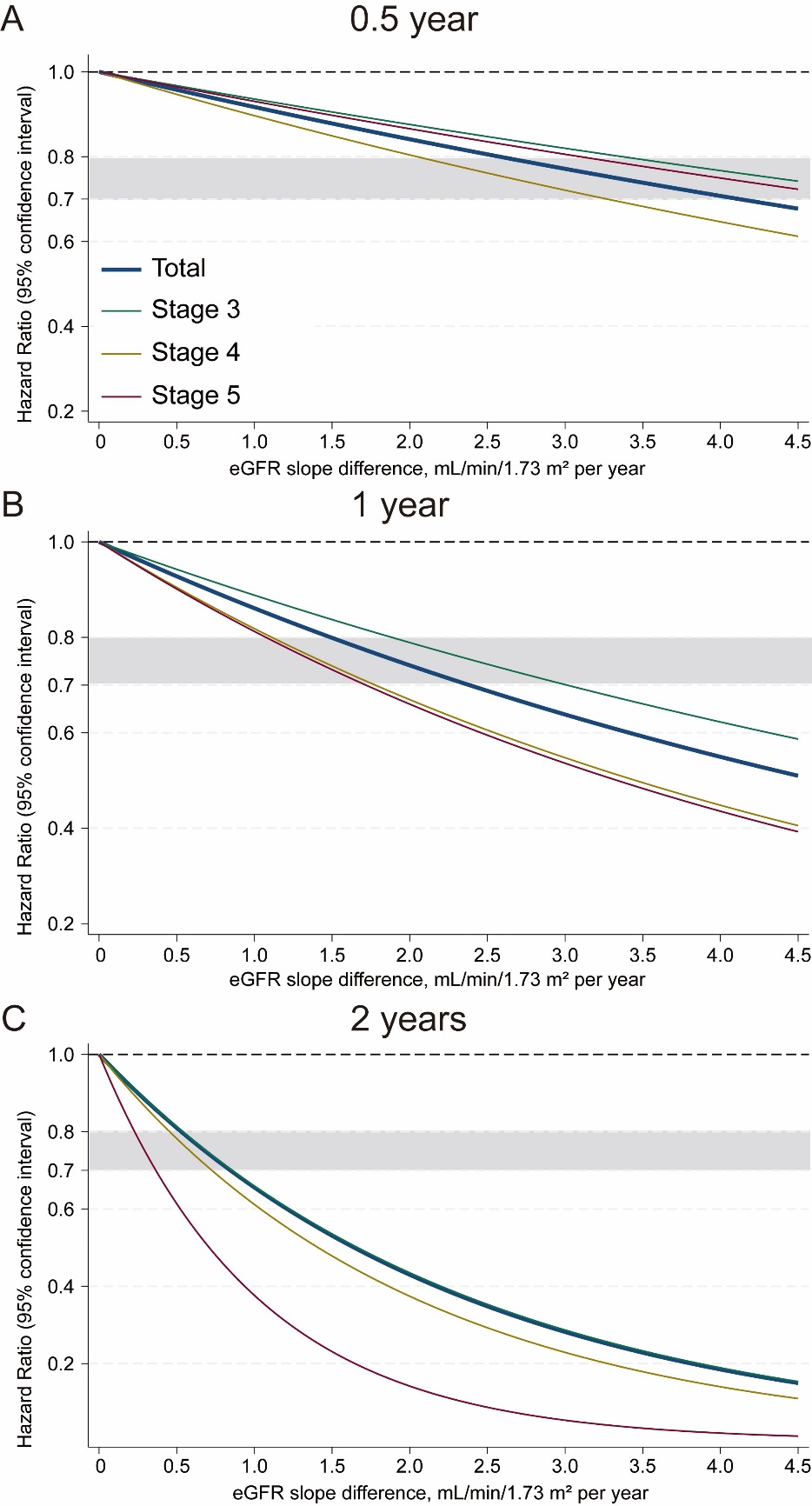


Figure S7. Estimated hazard ratios for KFRT associated with slower decline in eGFR slope across CKD stages and slope evaluation periods

Estimated HRs for subsequent KFRT are plotted against eGFR slope differences over 0.5 (**A**), 1 (**B**), and 2 years (**C**) in the total cohort and across CKD stages. Models were adjusted for age, sex, baseline eGFR, systolic blood pressure, CVD history, smoking status, and total cholesterol, in total participants and those with CKD stages 3, 4, and 5. Gray bands represent the HR between 0.7 and 0.8. Abbreviations: eGFR, estimated glomerular filtration rate; KFRT, kidney failure with replacement therapy; CKD, chronic kidney disease.
